# Towards Personalization in the Curative Treatment of Gastric Cancer

**DOI:** 10.3389/fonc.2020.614907

**Published:** 2020-11-30

**Authors:** Astrid E. Slagter, Marieke A. Vollebergh, Edwin P. M. Jansen, Johanna W. van Sandick, Annemieke Cats, Nicole C. T. van Grieken, Marcel Verheij

**Affiliations:** ^1^ Department of Radiation Oncology, Netherlands Cancer Institute, Amsterdam, Netherlands; ^2^ Department of Gastrointestinal Oncology, Netherlands Cancer Institute, Amsterdam, Netherlands; ^3^ Department of Surgery, Netherlands Cancer Institute, Amsterdam, Netherlands; ^4^ Department of Pathology, Amsterdam University Medical Centers, Amsterdam, Netherlands; ^5^ Department of Radiation Oncology, Radboud University Medical Center, Nijmegen, Netherlands

**Keywords:** gastric cancer, personalization, radiation oncology, multidisciplinary approach, future perspectives

## Abstract

Gastric cancer is the fifth most common cancer worldwide and has a high mortality rate. In the last decades, treatment strategy has shifted from an exclusive surgical approach to a multidisciplinary strategy. Treatment options for patients with resectable gastric cancer as recommended by different worldwide guidelines, include perioperative chemotherapy, pre- or postoperative chemoradiotherapy and postoperative chemotherapy. Although gastric cancer is a heterogeneous disease with respect to patient-, tumor-, and molecular characteristics, the current standard of care is still according to a one-size-fits-all approach. In this review, we discuss the background of the different treatment strategies in resectable gastric cancer including the current standard, the specific role of radiotherapy, and describe the current areas of research and potential strategies for personalization of therapy.

## Introduction

Gastric cancer remains a major health problem with worldwide over a million new cases per year (1). Gastric cancer is the third leading cause of cancer deaths worldwide, although it should be noted that there is a wide variation in incidence and mortality (1). The highest estimated incidence rates are seen in Eastern Asia, but also in Central and South America and in Central and Eastern Europe. By contrast, lower incidence rates are observed in Northern America, Northern Europe and Western Europe (1). Gastric cancer usually becomes symptomatic at an advanced stage, which is largely responsible for the poor outcome. In order to reduce gastric-cancer related mortality, both Japan and South-Korea have implemented screening programs, which have led to earlier detection of gastric cancer and improved survival rates (2–4).

Over the last decades, the management of patients with resectable gastric cancer has evolved from a complete surgical approach to a multidisciplinary strategy (5). Although multimodality treatment of gastric cancer patients is currently standard of care in all parts of the world, differences in type of standard (neo-)adjuvant treatment do exist frequently dictated by nationally developed and implemented guidelines. To illustrate, perioperative chemotherapy is the current standard of care in Europe (5), while postoperative chemotherapy is standard of care in most Asian countries (6–8). In the United States, both perioperative chemotherapy and preoperative chemoradiotherapy are recommended treatment strategies, of which perioperative chemotherapy is most frequently used (9).

Gastric cancer is a very heterogeneous disease. It can be subdivided according to tumor morphology, for which the Lauren classification is most widely used (10). More recently, molecular classifications have been introduced, including the classification by The Cancer Genome Atlas (11). Despite these insights, resectable gastric cancer is still being treated according to the one-size-fits-all principle. Since there are several effective and feasible options for (neo-)adjuvant treatment, this offers possibilities for personalization in gastric cancer management based on patient- and tumor characteristics. However, it is currently unknown which patients profit the most from what therapy.

In this review, we evaluate the background of the different multidisciplinary treatment approaches in resectable gastric cancer including the role of radiotherapy. In addition, possible options for personalization of therapy based on patient and tumor characteristics are explored.

## World-Wide Treatment Approaches

Current standard treatments differ over the world. **Table 1** provides an overview of the current treatment recommendations for (neo-)adjuvant treatments in various guidelines. The background and evidence for these guideline will be discussed below.

**Table 1:** Current treatment recommendations in different gastric cancer guidelines (5, 6, 8, 9).

Country/stage	Clinical stage IA	Clinical stage IB-IIIC
**United States**	Endoscopic or surgical resection	Resection with D2 lymph node dissection. Preferred strategy: perioperative chemotherapy (with fluorouracil and cisplatin, or fluoropyrimidine and oxaliplatin, or ECF/EOF/EOC/ECC, guideline was published in 2017, before results of the FLOT4-AIO).
**Europe**	Endoscopic or surgical resection	Resection with D2 lymph node dissection. Preferred strategy: perioperative chemotherapy with a platinum/fluoropyrimidine combination. Other postoperative pathways: postoperative chemoradiotherapy or postoperative chemotherapy
**Asia**	Endoscopic or surgical (with D1/D1+ lymph node dissection)	Resection with D2 lymph node dissection. Postoperative course depending on pathology stage: I: observation II/III: postoperative chemotherapy with S-1 monotherapy or oxaliplatin plus capecitabine*

*Guidelines in Asia differ slightly. In Japan, S-1 monotherapy is recommended for pathological stage II and capecitabine plus oxaliplatin for stage III. In Korea, both options are offered as treatment option. In China, combined chemotherapy with platinum and fluorouracil preferred (not exceeding 6 months) and for fragile patients fluorouracil monotherapy (not exceeding 12 months).

### The Surgical Approach

Until a few decades ago, surgery alone has been the only curative treatment in patients with non-metastatic resectable gastric cancer. However, despite improvements in surgical quality, prognosis of gastric cancer remains poor prognosis even in resectable disease (12, 13). The current surgical approach includes resection of the primary tumor with a generous margin plus extended D2 lymph node dissection (perigastric lymph nodes plus those along the left gastric, common hepatic and splenic arteries and the celiac trunk) (5). This “aggressive” surgical approach was first considered and investigated in Japan, where D2 lymph node dissection has been implemented in clinical practice a few decades ago. The survival results of this more extensive lymph node dissection were first published by Japanese surgeons in 1970 (14). The authors reported a small survival benefit among patients with pN0 disease, and a larger survival benefit in patients with pN+ disease with 5-year overall-survival (OS) rates increasing from 18% to 39%. In line with these results, another study reported that the more extended lymph node dissection led to an increase in 5-year OS from 33% to 58% for a patient group including both pN0 and pN+ disease (15). While the extended lymph node dissection had already been implemented in Asian countries, clinical trials in a Western population were awaited.

The first European trials showed increased postoperative mortality for patients who underwent a D2 lymph node dissection, which was therefore considered to be unsafe (16–18). Also, the first results of the Dutch D1D2 study were disappointing, and showed that patients who underwent D2 lymph node dissection had higher chance of postoperative complications and mortality (19). However, in the 15-year follow-up analysis of the D1D2 trial, D2 lymph node dissection was associated with a significant and persistent disease-specific survival benefit for patients who did not undergo splenectomy and/or removal of the pancreatic tail (20). The gastric-cancer-related deaths were higher in the D1 group as compared with the D2 group (48% vs. 37%). These observations were confirmed in a meta-analysis including 12 randomized controlled trials performed in both European and Asian countries, showing that a D2 lymph node dissection with spleen and pancreas preservation had a higher survival rate than less extensive lymphadenectomy in patients with resectable gastric cancer [Hazard Ratio (HR)= 0.65, 95% Confidence Interval (CI)= 0.52-0.80, p<0.001] (21). Currently, an extended D2 lymph node dissection with spleen and pancreatic gland preservation is also recommended by both European and American guidelines (5, 9). In South-America, NCCN guidelines or local guidelines are followed. In Australia, both the NCCN and ESMO guidelines are implemented in clinical practice.

### Differences in Multimodality Treatment Guidelines Between East and West

**Figure 1:**
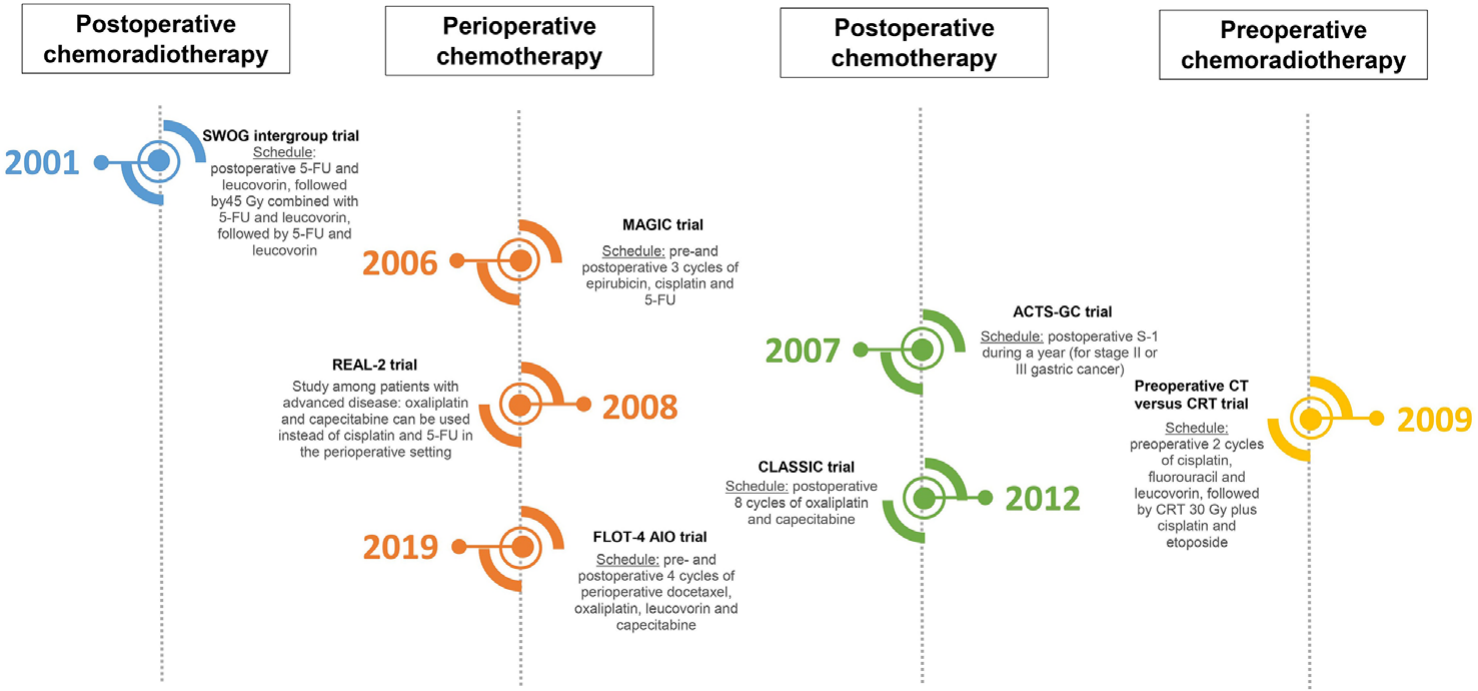
Timeline of different practice-changing randomized trials (26–31).

**Figure 2:**
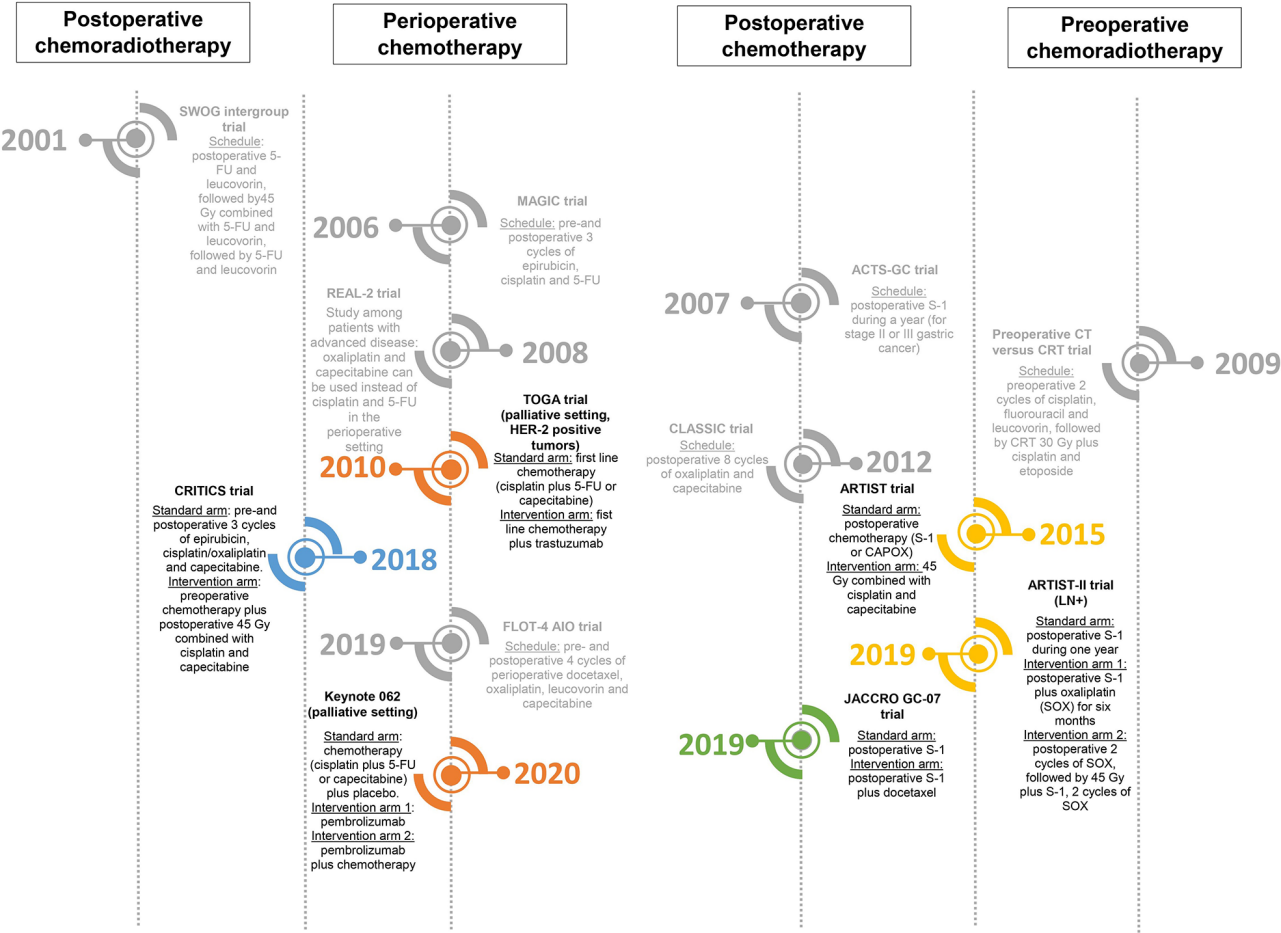
Timeline of different randomized trials that have not led to change of clinical practice (yet) in the curative setting, but have investigated important research questions and/or form the rationale behind ongoing (possibly practice changing) studies (46, 47, 49, 77, 90, 103).

The necessity for a multidisciplinary approach in the treatment of resectable gastric cancer (provided that the patient is fit enough) has been globally accepted. However, different guidelines are followed in different parts of the world. To understand the development of different approaches, it is important to note that patient- and tumor characteristics differ between East and West (22–25). Consequently, multidisciplinary (neo-)adjuvant treatments have been studied separately in different parts of the world. The studies which changed clinical practice and have led to the current standards are displayed in **Figure 1**; ongoing randomized phase II–III studies are shown in **Supplementary Table 1**. Studies which have investigated important research questions but have not led to change of clinical practice (yet) in the curative setting are displayed in **Figure 2**.

### Postoperative Chemoradiotherapy—West (United States and Europe)

The first positive study regarding (neo-)adjuvant treatment was the SWOG intergroup trial conducted in the United States (US) (32). This study enrolled patients from 1991 until 1998 and the final results were published in 2001. Eligible patients underwent a R0 resection and were randomized to postoperative observation versus postoperative chemoradiotherapy. Patients in the intervention group started with 5-fluouracil (5-FU) and leucovorin for 5 days, followed by chemoradiotherapy 28 days after initiating chemotherapy. Chemoradiotherapy consisted of 45 Gy in 25 fractions of 1.8 Gy, combined with an adapted dose of 5-FU and leucovorin on the first four and last 3 days of radiotherapy. One month after completion of radiotherapy, two 5-day cycles of 5-FU and leucovorin were given. After a median follow-up period of 5 years, the OS was 36 months in the intervention group, compared to 27 months in the observation group (p=0.006) with a HR for death of 1.52 (95% CI 1.23–1.52, p<0.001). Updated analyses demonstrated a persistent benefit from adjuvant chemoradiotherapy in patients with resectable gastric cancer (26). The SWOG-intergroup trial has often been criticized because only 10% of the study population underwent the advised extended (D2) lymph node dissection. It has been hypothesized that postoperative chemoradiotherapy was only effective in patients who underwent a limited (D1 or less) lymph node dissection and compensated for poor surgery. Furthermore, the chemoradiotherapy schedule used in the SWOG intergroup trial was associated with high rates of grades 3–4 toxicity. This high toxicity rate could have contributed to the limited implementation of postoperative chemoradiotherapy in Europe. Due to the concerns regarding toxicity, the chemotherapy regimen as used in the SWOG-intergroup trial is no longer in use in the United States (9). To optimize postoperative chemoradiotherapy regimens, several phase I–II trials have evaluated less toxic chemotherapy agents in combination with radiotherapy (33, 34).

### Preoperative Chemoradiotherapy—West (United States)

According to the National Comprehensive Cancer Network (NCCN) guideline that is adhered to in the United States, chemoradiotherapy can also be given preoperatively, next to perioperative chemotherapy (next paragraph) (9). This is in contrast to the European Society of Medical Oncology (ESMO) guideline in Europe, which does not recommend preoperative chemoradiotherapy as standard treatment (5). The first study on preoperative chemoradiotherapy in patients with resectable gastric cancer was published in 2001 (35). A total of 24 patients were treated with 45 Gy external beam radiotherapy with concurrent 5-FU. Resection was scheduled 4–6 weeks after chemoradiotherapy. Intraoperatively, patients received an additional 10 Gy. Most patients underwent gastric cancer resection with D2 lymphadenectomy (83%). Of the 19 resected patients, 12 (63%) had a major pathologic tumor responses, and two (11%) had a complete response. One of the concerns in the study was the relatively low number of resected lymph nodes. A comparison was made between the 19 resected patients in the study an similar time period patients treated with preoperative chemotherapy undergoing the same type of resection performed by the same surgeon. The number of resected lymph nodes was (both median as mean) lower in the patients receiving chemoradiotherapy.

A few other small phase II studies on preoperative chemoradiotherapy have been performed. In one of the first studies, 33 patients with localized gastric cancer (mainly T4 with or without nodal disease, M0) were treated with one cycle of chemotherapy, consisting 5-FU, leucovorin and cisplatin, followed by chemoradiotherapy (36). A total of 45 Gy was delivered in 25 fractions during 5 weeks, with concurrent 5-FU. A pathological complete response was achieved in 10 patients (30% of the assessable patients), and a partial response was noted in 8 patients (24% of the assessable patients). The 2-year OS rate was 54%. Another study performed by the same research group included 41 patients with localized gastric or gastroesophageal adenocarcinoma (mainly T3 disease with or without nodal disease) who were treated with two cycles of 5-FU with paclitaxel and cisplatin, followed by 45 Gy concurrent with 5-FU and paclitaxel (37). The pathological complete response rate was 20%, and the pathological partial response rate was 15%. The survival rate after more than 36 months of follow-up was 68%, which is very promising for this group of patients. In another phase II trial, 49 patients with stage IB-III gastric cancer were included. Patients received two cycles of 5-FU, leucovorin and cisplatin, followed by concurrent chemoradiotherapy consisting of 45 Gy with 5-FU and paclitaxel. A total of 83% of patients underwent resection. A pathologic complete response was confirmed in 11 (26%) out of 43 evaluable patients. The 1-year OS was 72% (38).

Other preoperative chemoradiotherapy schedules have been explored in Europe. One study explored the feasibility and efficacy of preoperative chemoradiotherapy consisting 25 fractions of 1.8 Gy in combination with carboplatin and paclitaxel in 25 patients with stage IB-IVA (M0) gastric cancer (39). Grade III adverse events were considered manageable: 12% gastrointestinal, 12% hematological and 8% other. The efficacy was encouraging with 40% (near) complete pathological responses.

A phase III study that compared preoperative chemotherapy with preoperative chemoradiotherapy in patients with stage T3-4/Nx/M0 gastro-esophageal junction (GEJ) tumors was performed in Germany (40). This study ran from 2,000 until 2005 and planned accrual for the first stage of the study was 200 patients. However, only 126 patients were randomized due to slow accrual. Patients in the chemotherapy arm received cisplatin, 5-FU and leucovorin with a total treatment time of 15 weeks, whereas patients in the chemoradiotherapy arm received the same type of chemotherapy during 12 weeks, followed by chemoradiotherapy. Concurrent chemoradiotherapy consisted of 30 Gy in fractions of 2 Gy, combined with cisplatin on days 1 and 8, and etoposide on days 3–5. Patients in the chemoradiotherapy group had more frequently a pathological complete response (16% vs. 2%) as well as tumor-free lymph nodes (64% vs. 38%). The 3-year OS rate was 28% in the chemotherapy group compared to 48% in the chemoradiotherapy group.

Preoperative chemoradiotherapy has been indicated in the NCCN flow-chart as possible treatment for patients with resectable gastric cancer (based on level 2B evidence, meaning that this statement is based upon lower-level evidence, but with NCCN consensus that this intervention is appropriate) (9). The results of small studies evaluating preoperative chemoradiotherapy are promising in terms of efficacy. Large randomized controlled trials are needed to compare toxicity and response rates between preoperative chemoradiotherapy and preoperative chemotherapy. Currently, these large randomized controlled trials are lacking, which is probably the reason that preoperative chemoradiotherapy has not been mentioned as treatment option in the European guideline (5).

### Perioperative Chemotherapy—West (Europe and United States)

While the SWOG-intergroup trial was running in the United States, the MAGIC trial enrolled patients in the United Kingdom (UK) between 1994 and 2002 (27). The final results were published in 2006. A total of 503 patients were randomized to undergo surgery alone versus perioperative chemotherapy. Perioperative chemotherapy included three cycles of epirubicin, cisplatin and 5-FU. The OS increased in the perioperative chemotherapy group, with a 5-year OS of 36% compared to 23% in the surgery only group. The HR for progression-free survival (PFS) was 0.66 (95% CI 0.53–0.81, p<0.001).

The benefits of the preoperative part of perioperative treatment has been confirmed in a meta-analysis, including studies from 1990 to 2012 (41). Twelve comparable studies were evaluated including 1,566 patients with GEJ or gastric cancer, using several preoperative chemotherapy schedules. Preoperative chemotherapy resulted in a higher chance of obtaining an R0 resection with an odds-ratio (OR) of 1.38 (95% CI 1.08-1.78, p=0.01). In addition, preoperative chemotherapy increased the likelihood of down-staging, with an OR of 1.71 (95% CI 1.26–2.33, p<0.001). Also, survival improved with an OR of 1.32 (95%CI 1.07–1.64, p=0.01).

Several studies have been conducted since the MAGIC trial. The chemotherapy combination of epirubicin, cisplatin and 5-FU was already widely used in patients with advanced and/or metastatic esophagogastric cancer. The combination of chemotherapy has been further explored in the REAL-2 trial, in which oxaliplatin and capecitabine were considered as alternatives for cisplatin and 5-FU (28). The study had a two-by-two design, and 1,002 patients with advanced and/or metastatic esophagogastric cancer were randomized to triplet chemotherapy with epirubicin, cisplatin and either 5-FU (ECF) versus capecitabine (ECC), or triplet chemotherapy with epirubicin and oxaliplatin plus either 5-FU (EOF) or capecitabine (EOC). Median survival rates at 1 year for ECF, ECC, EOF, and EOC were 38%, 41%, 40%, and 47%, respectively. Toxicity profile was similar for capecitabine and 5-FU. Compared with cisplatin, oxaliplatin was associated with lower incidences of grades 3–4 neutropenia, renal toxicity and thromboembolism and slightly higher incidences of diarrhea and neuropathy. It was concluded that capecitabine was as effective as 5-FU and that oxaliplatin was a good alternative for cisplatin. Although this study was performed in patients with esophagogastric cancer in the palliative setting, this resulted in adaptation of the gastric cancer guidelines in Europe, which allowed oxaliplatin and capecitabine as alternative for cisplatin and 5-FU in the curative setting (5).

Between 2010 and 2015, the FLOT4-AIO study was performed in Germany, of which the results were presented at the ESMO annual meeting in 2017 (42). A total of 716 patients with at least cT2, any N gastric cancer or cancer of the gastro-esophageal junction (GEJ) were allocated to receive either perioperative three cycles of 3-weekly epirubicin, cisplatin, and capecitabine/5-FU (ECC/ECF) or perioperative two cycles of 2-weekly docetaxel, leucovorin, oxaliplatin and 5-FU (FLOT). As shown in a pathology analysis in the phase II part of the study, a complete pathological response was observed in 16% of patients in the FLOT group compared to 6% in the ECC/ECF group (p=0.02) (43). Overall-survival was higher in the FLOT group than in the ECC/ECF group with a HR of 0.77 (95% CI 0.63–0.94; median OS 50 versus 36 months), whereas the long-term outcomes in the ECC/ECF arm were comparable with those in the MAGIC trial (27). The results of the FLOT-AIO4 trial provided a next step forward and the treatment regimen was implemented in many European countries and in the United States before the final publication came out in 2019 (29). Perioperative chemotherapy is currently the preferred treatment choice in both Europe and the United States.

### Postoperative Chemotherapy—East

Slightly later than the SWOG-intergroup trial and the MAGIC trial, the ACTS-Gastric Cancer (GC) trial was running in Japan (44). The ACTS-GC trial enrolled patients from 2001 to 2004, and the final results were published in 2007. During the study period, the screening program was already implemented in Japan (2). Eligible patients had stage II-III gastric cancer and underwent R0 surgery with D2 lymph node dissection. A total of 1059 patients were randomized to observation only versus postoperative chemotherapy consisting of S-1 during 1 year. Three-year OS was 70% in the control group as compared to 80% in the intervention (S-1) group. The HR for death in de S-1 group was 0.68 (95% CI 0.52–0.87, p=0.003).

The CLASSIC trial was another study investigating the role of postoperative chemotherapy and enrolled patients from China, South-Korea and Taiwan between 2006 and 2009 (30, 31). A total of 1035 patients with stage II-III gastric cancer who underwent an R0 resection with D2 lymph node dissection were randomized to postoperative observation only or postoperative chemotherapy. Postoperative chemotherapy included eight cycles of capecitabine and oxaliplatin (CAPOX). The 3-year disease-free survival was 74% in the CAPOX group, compared to 59% in the observation only group, with an HR of 0.56 (95% CI 0.44–0.72; p<0.001).

Based on the results of the ACTS-GC and the CLASSIC trials, the Japanese guidelines recommend adjuvant S-1 monotherapy for patients with stage II disease, and S-1 monotherapy or an oxaliplatin-containing chemotherapy combination such as CAPOX in stage III disease (7). In South-Korea, both S-1 and CAPOX are offered as postoperative treatment options for the same group of patients (8). Combined postoperative chemotherapy, such as CAPOX, is recommended in China for stage II-III gastric cancer (6). In Western countries, several negative studies have been published on the survival benefit of postoperative chemotherapy. Therefore, the role of postoperative chemotherapy without preoperative chemotherapy in very limited in Western countries (45). Recently, the interim analysis of the Japanese JACCRO GC-07 trial has been published (46). A total of 915 patients with stage III gastric cancer who underwent an R0 resection with D2 lymph node dissection were randomized to postoperative S-1 versus postoperative docetaxel plus S-1. After a median follow-up of 12.5 months, the results revealed a superior recurrence-free survival (RFS) in the docetaxel plus S-1 group, with a 3-year RFS of 66% versus 50% (HR 0.632, 99.99% CI 0.400–0.998, p<0.001). Grade 3 toxicities, in particular hematological, were higher in the docetaxel plus S-1 group, but were considered manageable. It is most likely that the use of S-1 plus docetaxel will be implemented for stage III gastric cancer in the Asian guidelines in the near future.

## Chemoradiotherapy as Part of Multimodality Treatment—Current Knowledge and Research Areas

Several studies have established the added value of (neo-)adjuvant chemoradiotherapy over surgery alone. The optimal regimen for chemoradiotherapy, however, has not been identified yet. In this paragraph we will discuss research areas in both pre- and postoperative chemoradiotherapy and future perspectives on personalization of the radiotherapy component.

### Chemoradiotherapy in the Postoperative Setting

In the Korean ARTIST trial (2004–2008) postoperative chemotherapy was compared to postoperative chemotherapy in combination with chemoradiotherapy in 458 patients who underwent an R0 resection with D2 lymph node dissection (47, 48). Postoperative chemotherapy consisted of six cycles of cisplatin and capecitabine, while postoperative chemoradiotherapy consisted of two cycles of cisplatin and capecitabine followed by 45 Gy in 25 fractions of 1.8 Gy with concurrent capecitabine, again followed by two cycles of cisplatin and capecitabine (48). Compliance rates were 75% for the chemotherapy arm compared to 82% of the chemotherapy plus chemoradiotherapy combination arm. The estimated 3-year RFS was 74% in the chemotherapy group compared to 78% in the chemoradiotherapy group (p=0.862). However, in planned multivariate analysis, the chemoradiotherapy regimen showed prolonged RFS after adjustment for stage in the lymph node positive group (3-year RFS 72% in the chemotherapy arm compared to 76% in the chemoradiotherapy arm; p=0.04). Based on this latter observation, the ARTIST-II trial was designed to investigate the added value of chemoradiotherapy in nodal positive patients, of which the final results are not published yet.

Similar to the ARTIST trial, the CRITICS trial focused on postoperative chemoradiotherapy, although this trial had been designed from a European perspective. Integrating the regimens from both the MAGIC and the SWOG-intergroup trial, the CRITICS study aimed to combine optimal loco-regional and systemic treatment (49). Patients from the Netherlands, Sweden and Denmark with stage IB-IVA (M0) GEJ or gastric cancer were upfront (before any treatment) randomized between perioperative chemotherapy (comparable to MAGIC trial) and preoperative chemotherapy plus postoperative chemoradiotherapy. Both preoperative as postoperative chemotherapy consisted of three cycles of 3-weekly epirubicin, cisplatin or oxaliplatin, and capecitabine. The chemoradiotherapy schedule used in the CRITICS trial consisted of 45 Gy in 25 fractions of 1.8 Gy, combined with weekly cisplatin and daily capecitabine. Between 2007 and 2015, a total of 788 patients was included. Compliance rates were 46% for the chemotherapy arm compared to 50% for the chemoradiotherapy arm. After a median follow-up of 61 months, there was no difference in median overall survival (HR 1.01, 95% CI 0.84–1.22, p=0.90), or the 5-year survival rate (42% vs. 40%, chemotherapy and chemoradiotherapy group respectively). Subgroup analyses are currently being performed in order to identify subgroups of patients who might benefit from one of these treatment options. The compliance rate in the CRITICS trial was lower than in the ARTIST trial, probably mainly due to the design of the study. Also, survival rates were different between the studies. Patients in the ARTIST trial underwent surgery for less advanced stage (60% had stage IB or II disease) compared to the CRITICS trial, in which the majority of the patients had advanced disease.

### Chemoradiotherapy in the Preoperative Setting

The added value of preoperative chemoradiotherapy has not yet been derived from comparative trials. However, several trials are underway in different parts of the world generating new results.

One of the ongoing randomized phase-III studies is the TOPGEAR trial, which was designed in Australia but is also recruiting patients from several countries in Europe and Canada (50). Patients are randomized to either perioperative chemotherapy or preoperative chemoradiotherapy plus perioperative chemotherapy. In the perioperative chemotherapy arm, patients receive three cycles of epirubicin, cisplatin, and capecitabine or 5-FU (ECC/ECF) before and after surgery. In the preoperative chemoradiotherapy plus perioperative chemotherapy arm, patients are treated preoperatively with two cycles of ECC/ECF followed by chemoradiotherapy, and postoperatively with three cycles of ECC/ECF. Chemoradiotherapy consists of 45 Gy in 25 fractions, combined with daily 5-FU or capecitabine throughout the entire radiotherapy period. Since the final publication of the favorable results of the FLOT-AIO4 trial, the FLOT regimen is allowed as replacement for ECC/ECF in this trial. The interim toxicity data of the TOPGEAR trial have been published in 2017, showing that preoperative chemoradiotherapy did not increase preoperative toxicity compared to the standard arm. Preoperative chemoradiotherapy is therefore considered safe and feasible. The primary endpoint of the study is OS and the final results of the study are awaited.

Similar to the TOPGEAR trial, the CRITICS-II trial is investigating the role of preoperative chemoradiotherapy (51). This trial has been designed to identify an optimal preoperative treatment regimen, without any postoperative treatment. One of the major problems in patients undergoing perioperative treatment is the postoperative treatment compliance, which is only around 50% of the patients who have started preoperative treatment. It is hypothesized that by intensifying the preoperative part of the treatment, the postoperative treatment part can be safely omitted. Patients with stage IB-IIIC gastric cancer are randomized to one of three arms (1): preoperative chemotherapy (2), preoperative chemotherapy plus chemoradiotherapy, or (3) preoperative chemoradiotherapy. Patients randomized to the chemotherapy arm receive four cycles of docetaxel, oxaliplatin, and capecitabine (DOC); patients randomized to chemotherapy plus chemoradiotherapy receive two cycles of DOC, followed by chemoradiotherapy consisting of 45 Gy in 25 fractions, in combination with weekly paclitaxel and carboplatin; and patients randomized to chemoradiotherapy receive only 45 Gy of radiation in 25 fractions, in combination with weekly paclitaxel and carboplatin. The primary endpoint of the study is 1-year event-free survival (EFS). To our knowledge, this is the first and currently the only running comparative trial completely focusing on the preoperative (neoadjuvant) treatment regimen.

One of the concerns of preoperative chemoradiotherapy is that it might increase postoperative complications. Interim results of the TOPGEAR trial did not show an increased incidence in postoperative complications in the chemoradiotherapy group as compared with the chemotherapy group (50). Preoperative chemoradiotherapy is mentioned in the NCCN guidelines as treatment option in patients with resectable gastric cancer (9). Therefore, in the US there is more experience with chemoradiotherapy for gastric cancer patients compared to Europe or Asia. In 2017, a retrospective analysis of the MD Anderson Cancer Center was published including 346 patients with resectable gastric cancer, of whom 44% underwent preoperative chemoradiotherapy. There was no association between type of preoperative therapy and the risk of anastomotic leakage (52). These results support the notion that neo-adjuvant chemoradiotherapy does not increase the postoperative complication rate.

### Individualization of Radiotherapy Treatment

Similar to the chemotherapy part of treatment, also in defining radiation fields (Clinical Target Volume, CTV) a one-size-fits-all strategy is used. The CTV is mainly based on the location of the primary tumor, lymph node metastasis pattern and in the postoperative setting on surgical anastomoses. To improve inter-observer variations, especially in clinical trials, contouring atlases have been created (53). Future elaborate studies on recurrence patterns after radiotherapy are needed to personalize CTV’s, based on patient characteristics, but probably also on genomic parameters. It is hypothesized that preoperative radiotherapy enables better delineation of the CTV because of the non-disturbed anatomy, but this has to be proven. Above that, the daily variation in size and position of the CTV can be large. Novel techniques like MRLinac based radiotherapy and “library of plans” planning techniques can probably address this (54).

## Personalization of Multimodality Treatment

In the following paragraphs we explore potential strategies for personalization of multimodality treatment. Personalization of therapy could be addressed from different perspectives: clinical factors, tumor characteristics known before treatment, or tumor/patient characteristics known after surgery.

## Personalization of Multimodality Treatment—Clinical Factors

Several clinical factors should be taken into consideration in the multidisciplinary treatment of patients with resectable gastric cancer. In this paragraph, we will discuss the impact of (biological) age and gender.

### Age

Older age has a significant impact on the management of patients with gastric cancer. As investigated in a German population-based study, around 60% of the total gastric cancer population had an age of 70 years or higher at time of diagnosis and older patients less frequently underwent surgery (55).

Older patients are generally less fit than younger patients due to comorbidity, and are more likely to experience side-effects during chemo(radio)therapy. The only study performed in the curative setting, is a subgroup analysis of the CRITICS trial, in which older patients were defined as those individuals aged 70 years or older at time of inclusion (56). In older patients, the incidence of severe toxicity was higher during preoperative chemotherapy (77% vs. 62%, p<0.001). Nevertheless, curative surgery was performed in the same proportion of older patients compared to younger patients. Postoperatively, there were no significant differences in toxicity, although older patients started postoperative chemotherapy with reduced dose. For patients who started postoperative chemoradiotherapy, there were no differences in completion of therapy. It is of note that patients who started postoperative chemoradiotherapy form a highly selected group. Therefore, it is uncertain if this conclusion can be projected to the broader population. Although this subgroup analysis suggests more toxicity, the results also indicate that fit older patients should not be excluded a prior from a multimodality treatment.

The North Central Cancer Treatment Group (NCCTG) performed a pooled analysis of eight clinical trials including 367 patients with metastatic esophagogastric cancer (57). In this study, older patients were defined as those individuals with an age of 65 or higher. Not surprisingly, older patients had a reduced performance status (PS) compared to younger patients (PS 2-3: 19% vs. 8%, p<0.001). Also, severe toxicity was more common in patients with a higher age (73% vs. 66%, p=0.02). The higher rate of severe toxicity in older patients was mainly caused by the difference in neutropenia, fatigue, infection, stomatitis, renal failure, and hypotension.

It could be hypothesized that it would be better to treat older patients with reduced doses of chemotherapy. This should preferable not be at the cost of a reduced survival benefit. To our knowledge, only two studies investigated chemotherapy dose reduction in older patients with advanced gastric cancer. One study from the UK included 541 patients who were unable to receive full doses of perioperative epirubicin, cisplatin, and capecitabine because of age or frailty (58). Instead, they were 1:1:1 treated with level A dose (oxaliplatin 130 mg/m^2^ on day 1 and capecitabine 625 mg/m^2^ on days 1–21, every 21 days); level B dose (80% of level A doses); or level C dose (60% of level A doses). Preliminary data showed that patients who received the lowest dose had the lowest incidence of severe toxicity. Interestingly, the PFS in level C was non-inferior compared to the PFS in levels A and B. The FLOT65+ trial, also from the UK, investigated reduced dosing in older patients treated with the FLOT regimen (59). Patients aged 65 years or older with esophagogastric cancer in the palliative setting were eligible. In total 143 patients were randomized to receive 5-FU, leucovorin and oxaliplatin (FLO) with docetaxel (FLOT) or without docetaxel (FLO). The primary endpoint of this study was feasibility and tolerability. The incidence of severe toxicity was higher in the FLOT group than in the FLO group (82% vs. 39%, p<0.001). Although treatment duration was comparable in both groups, the addition of docetaxel did not seem to give any PFS benefit in patients older than 70 years (p=0.65).

Not only age should be considered as risk factor for higher frequency of toxicity or worse outcome; also, comorbidity should be taken into account. A Japanese study confirmed that comorbidity is a risk factor for poor survival (60). To indicate the severity of comorbidity, the Charlson Comorbidity Index (CCI) was used, with a higher score indicating that patients had more comorbidities. The HR for all-cause mortality per point CCI increase was 1.12 (95% CI 1.02–1.23). The results of this study indicate that also comorbidity (frailty/biological age) should be taken into account when making treatment decisions.

In conclusion, if fit enough to undergo (neo-)adjuvant treatment, patients should not be excluded from a multimodality treatment solely based on age. Currently, international guidelines for the management of patients with gastric cancer do not provide recommendations for chemo(radio)therapy dose based on (biological) age. It is unknown whether older patients have the same needs compared to younger patients, or that a modified combination or adapted dose would be more appropriate. Based on the current evidence, clinical practice should not be adapted based on age alone. Future trials are needed for this group of patients.

### Gender

Gender is not often considered as a potential factor to individualize therapy. However, gender has been reported to influence treatment-related toxicity and impacts on outcome. Men are more often diagnosed with gastric cancer compared to women, and constitute two-thirds of the gastric cancer population (1). Not only the incidence is different for men and women, there are also gender differences in tumor subtypes. For example, gastric cancer associated with MSI-high is more common in female patients (OR 1.57, 95% CI 1.13–2.20; p<0.001), as demonstrated in a systematic review including 18,612 patients (61). A pooled analysis among 70 studies with 16,952 cases showed that the incidence of EBV positivity was twice as high in men compared to women (62). In the following paragraphs, we will discuss individualization of therapy based on tumor characteristics.

Besides differences in tumor types between men and women, there also seems to be a variation in treatment related toxicity, although not studied very widely. A pooled analysis of data from four randomized trials in patients with advanced esophagogastric cancer (non-curative setting) included a total of 1,654 patients, of whom the majority was male (80%) (63). All patients in this analysis were treated with first-line chemotherapy, included studies incorporated at least one arm consisting of a platinum/fluoropyrimidine/anthracycline triplet chemotherapy regimen. The occurrence of at least one Serious Adverse Event (SAE) was 45% in women compared to 36% in men (p=0.012). Especially the risk of gastrointestinal toxicity (adjusted for potential confounding factors) was enhanced (OR 1.50, 95% CI 1.07–2.12). Apart from gender-related variation in terms of toxicity, there might be a trend towards better survival in female patients. In the previously mentioned pooled study, multivariate survival analysis showed a better OS for female patients (HR 0.83, 95% CI 0.72–0.96), p=0.011). Another pooled analysis among 3265 patients with gastric cancer in the curative setting showed comparable results (64). Female patients experienced more severe nausea (12 vs. 7%, p=0.006), vomiting (10% vs. 5%), p<0.001), and diarrhea (9% vs. 4%, p=0.001), but were also more likely to achieve a complete/near complete response (p=0.002) with HR of both RFS and OS was 0.78 (p<0.001). The above results raises the question whether doses should be reduced in female patients because of the enhanced risk of severe toxicity, or the opposite: are men currently being under-dosed? Little data is available on pharmacokinetics and pharmacodynamics (PK-PD) differences between males and females. In some studies, a lower clearance of fluorouracil has been found in female patients (65, 66). More research is necessary to investigate whether there are PK-PD differences between men and women, and whether dose adaptation based on gender is appropriate.

## Personalization of Multimodality Treatment—Tumor Characteristics Known Before Treatment

Several tumor related factors could be used to individualize therapy in the future. In this paragraph we will discuss several factors known before surgery: Lauren classification, HER-2 overexpression, EBV associated-, and MSS/MSI-high tumors.

### Lauren Classification

Gastric cancer can be subdivided according to the Lauren classification (10). This is the oldest and in clinical practice the most frequently used classification of gastric adenocarcinoma. It divides gastric cancer into two subtypes: the intestinal type and the diffuse type. Patients with the intestinal type of gastric cancer are more frequently male and have an older age, while the diffuse type of gastric cancer is more common in younger women (67, 68). Currently, no distinction according to Lauren classification is being made in the recommendations of (neo-)adjuvant treatment of patients with resectable gastric cancer, although it is known that intestinal and diffuse type gastric cancer respond differently on treatment. In general, patients with diffuse type gastric cancer have a lower response rate after preoperative treatment. A large study, which used data of the AGAMENON registry (mainly Spanish centers), investigated the objective response rate (radiologically) among the different subtypes of gastric cancer in over 1,300 cases (mainly patients with metastases) treated with doublet or triplet chemotherapy. Patients with indeterminate tumors were excluded from this analysis. The authors concluded that the diffuse type of gastric cancer was associated with a lower response rate, compared to the intestinal type (HR 0.719, 95% CI 0.525–0.987, p=0.039) (69). One of the limitations was the lack of central pathological and radiological revision. A side-study from the FLOT4-AIO study has been performed, which also showed that intestinal type gastric cancer achieved more frequently a pathologic complete response (23%) compared to diffuse type (10%) on preoperative treatment with FLOT (43). In addition, patients with diffuse type gastric cancer have a poorer OS compared to patients with intestinal type (69–71).

In the future, Lauren classification might be one of the important characteristics to individualize treatment. To our knowledge, there is currently no data available on response differences on chemoradiotherapy between intestinal and diffuse type gastric cancer. In esophageal cancer, which is often treated with preoperative chemoradiotherapy, a pathological (near) complete response was observed in 24% of the diffuse type cancers compared to 60% in the intestinal type cancers (p=0.015) (72). Future research is needed to identify the optimal treatment approach for gastric cancer according to histological subtype.

### Human Epidermal Growth Factor Receptor 2 (HER-2) Overexpression/Amplification

The mean reported percentage of gastric cancers with HER-2 overexpression lies around 18% (73) and has been associated with intestinal type gastric cancer (74). HER-2 overexpression has a negative impact on survival, as shown in two meta-analyses (74, 75).

HER-2 amplification/overexpression might also be a good target for personalized treatment in the curative setting. HER-2 gives a strong proliferative signal and its overexpression on tumor cells subsequently enhances this effect, and is therefore an excellent candidate for targeted therapy (76). In the metastatic setting personalization based on HER-2 overexpression is already standard of care based on a large worldwide phase III study (TOGA trial). In this trial conducted in patients with EGJ or gastric cancer (77), the primary objective was to assess the clinical efficacy and safety of trastuzumab added to chemotherapy as first-line treatment. A total of 594 patients were randomized between chemotherapy (fluorouracil or capecitabine in combination with cisplatin) with trastuzumab or chemotherapy without trastuzumab. The OS was significantly higher in patients receiving trastuzumab as compared to those who did not receive trastuzumab, with an HR of 0.74 (95% CI 0.60–0.91, p=0.0046).

An example of a study in the curative setting is the PERTRARCA study by the FLOT-AIO group, of which the results were recently presented at the ASCO annual meeting (78). Patients with resectable HER-2 positive esophagogastric carcinoma were randomized between four perioperative cycles of FLOT with or without trastuzumab, followed by nine cycles of trastuzumab plus pertuzumab. A total of 81 patients were randomized. Patients treated with FLOT plus trastuzumab/pertuzumab had a significantly higher change of achieving a complete pathological response (25% vs. 12%, p=0.02). In addition, significantly more patients in the FLOT plus trastuzumab/pertuzumab group had pathological tumor-negative lymph nodes (68% versus 39%). The RFS slightly increased in the FLOT plus trastuzumab/pertuzumab group compared to the FLOT only group (HR 0.58, p=0.14). These promising results, however, were at the price of higher rates of diarrhea (41% vs. 5%) and leukopenia (23% vs. 13%).

Another study, which is ongoing, includes HER-2 positive GEJ or gastric cancer patients and randomizes between preoperative chemotherapy (three cycles of cisplatin and capecitabine or 5-FU) versus preoperative chemotherapy plus trastuzumab versus preoperative chemotherapy plus trastuzumab and pertuzumab (INNOVATION trial, NCT 02205047). The results of this study are not yet available.

Especially nivolumab and trastuzumab are currently being investigated in the curative setting. In addition, multiple new HER-2 targeting agents are currently being studied. One of these compounds is deruxtecan, an antibody drug conjugate which has shown very promising results in patients with gastric cancer in the metastatic setting (79).

In summary, treatment with targeted agents is a new therapeutic approach in patients with gastric cancer in the palliative setting and preliminary results are encouraging also for the curative setting.

### Epstein-Barr Virus (EBV) and MSS/MSI-High

In 2014, as part of The Cancer Genome Atlas project, 295 gastric adenocarcinomas were extensively molecularly characterized resulting in four different subtypes: the chromosomal instability (CIN), genomically stable (GS), microsatellite instability (MSI), and Epstein-Barr virus (EBV) positive subtype, of which the most common subtypes are CIN and GS (11).

Epstein-Barr Virus associated gastric cancer accounts for 7%–8% of the total gastric cancer population, but the reported incidence varies widely (80). There is no firm association between presence of EBV and response to chemotherapy (81), although it does seem associated with a better prognosis with respect to OS (82). All EBV-associated tumors express viral proteins, which contribute to malignant transformation (83). EBV-associated tumors have robust programmed death-ligant-1 (PDL-1) expression, making them potentially targets for immunotherapy (84). Immune checkpoint regulators are expressed on the surface of immune cells; immune checkpoint inhibitors bind to the PD-1 receptor and blocks the interaction between the PD-1 receptor and PDL-1. This action inhibits T-cell proliferation and secretion of cytokines, which enables anti-tumor response (85, 86).

Small studies have been conducted using immunotherapy for metastatic gastric cancer patients, showing very promising results for EBV-positive tumors (stable disease or better response in 90%–100%) (87, 88). This indicates that EBV positivity may be an important predictive biomarker for treatment with immunotherapy. Since the observation that patients with advanced mismatch repair-deficient cancers resulting in microsatellite instability (MSI), had a good response on immune-checkpoint blockade regardless of tumor type (89), this feature has also been extensively studied within gastric cancer patients. In the metastatic setting, anti PDL-1 treatment with pembrolizumab resulted in increased OS (exploratory analysis among 33 patients with MSI-high tumors, 1-year OS 79% versus 47%, HR 0.29 (95% CI 0.11–0.81) for treatment with pembrolizumab versus chemotherapy) (phase III study) (90).

MSI-high tumor accounts for around 22% (rage 12%–34%) of gastric cancer cases (45). Microsatellite instability is characterized by the inability to repair microsatellite regions from defects in the DNA mismatch repair system, which is responsible for the surveillance and correction of DNA replication (45).

In a subgroup analysis of the MAGIC trial (27), MSI results were available for 303 out of 503 patients (91). The vast majority of the patients had microsatellite stable (MSS) tumors (n=283 93%), while 20 (7%) patients had MSI-high tumors. A total of 19 patients were included in the OS analysis, of whom 10 were treated with surgery only and nine patients with perioperative chemotherapy. Interestingly, patients treated with surgery only showed better OS compared to the patients who received perioperative chemotherapy (HR 0.42, 95% CI 0.15–1.15, p=0.09). Although the number of this subgroup analysis was limited, the results of this analysis are potentially impactful and warrant further investigation. Also the CLASSIC trial—in which patients were treated with surgery alone or with postoperative chemotherapy—performed a post-hoc analysis (92). Of the 592 patients, 40 patients had MSI-high tumors. There was no survival benefit in the patients with MSI-high tumors with a 5-year RFS of 84% versus 86% (p=0.931). Kohlruss et al. investigated the predictive value of MSI-high tumors, showing that MSI-high tumors were not associated with response (81). Notable is that the number of patients with MSI-high tumors who were treated with preoperative chemotherapy and of whom response assessment was available was only 15. Based on these studies, patients with MSI-high gastric tumors might not benefit from chemotherapy. One meta-analysis is available including data from the MAGIC, CLASSIC, ARTIST and ITACA-S trials (93), which concluded that gastric cancer patients with MSI-high tumors do not benefit from chemotherapy (94).

The place of immunotherapy in patients with MSI-high gastric cancer in the curative setting is currently unknown and part of the research question of multiple ongoing clinical trials both in the entire gastric cancer population as in a subset of gastric cancer patients. An overview of all trials in different phases in the curative setting of gastric cancer treatment, including immunotherapy and targeted therapy, are displayed in **Supplementary Table 2**.

## Personalization of Multimodality Treatment—Tumor/Patient Characteristics Known After Surgery

### Tumor Regression

Patients with significant residual disease after preoperative therapy have a poorer prognosis compared to good responders (95–97). There are several scoring systems to classify tumor regression, of which the Mandard score (98) and the Becker score (99) are the most widely used. Intuitively, it is unlikely that patients with a poor tumor response on preoperative chemotherapy would gain survival benefit from repeating the same chemotherapy regimen postoperatively. Because a poor response on preoperative therapy is also an prognostic unfavorable factor (100), it is a methodological challenge to retrospectively investigate whether those patients benefit from identical postoperative chemotherapy. This challenging research question has been investigated by a research group from the UK (101). The study included patients with resectable gastric cancer who were all intended to receive perioperative chemotherapy. Survival was studied for patients who received the postoperative part of treatment and those who did not (due to several reasons, of which the most common was delay due to postoperative complications). Although the groups were not completely comparable (higher age, higher Clavien-Dindo (scoring system for postoperative complications) and lower T-stage in patients who did not receive postoperative treatment), administration of postoperative chemotherapy did not lead to a survival benefit. We do not know whether postoperative chemoradiotherapy is a more appropriate postoperative regimen for poor responders. The best treatment strategy towards these patients is unknown and warrants further investigation.

### Lymph Node Positive Disease

Patients with tumor-positive lymph nodes at time of resection form another challenge. As earlier discussed, for patients with resectable gastric cancer who were included in the ARTIST trial, postoperative chemoradiotherapy in addition to postoperative chemotherapy seemed to have a beneficial effect only in the lymph node positive patient group (48). In a subgroup analysis including patients with lymph node positive disease, it was shown that patients with a higher lymph node ratio had a worse RFS. Interestingly, the beneficial effect of the addition of postoperative chemoradiotherapy to postoperative chemotherapy was more pronounced in the patients with a high lymph node ratio (>25%) compared to those with a low lymph node ratio (102). In 2019, the (interim) results of the ARTIST-II trial were presented at the ASCO annual meeting. A total of 538 from the planned 900 patients with stage II/III, lymph node positive gastric cancer were 1:1:1 randomized to receive S-1 for 1 year, S-1 plus oxaliplatin (SOX) for 6 months, or SOX plus chemoradiotherapy. SOX plus chemoradiotherapy included two cycles of SOX, followed by S-1 daily combined with 45 Gy in 5 weeks, followed by four cycles of SOX. The 3-year RFS rates were 65%, 78%, and 73% for S-1, SOX and SOX plus chemoradiotherapy, respectively. No difference in RFS was documented between SOX and SOX plus chemoradiotherapy (HR 0.910, p=0.667), leading to the conclusion that both SOX and SOX plus chemoradiotherapy were effective in prolonging RFS, compared to S-1 monotherapy (103). Treatment was generally well tolerated in both arms. For more details, the final publications is awaited. Thus, intensification of treatment regimens for patients with lymph node positive disease can lead to prolongation of RFS. The final results of the ARTIST-II trial have to be awaited. There might, also in the perioperative setting, be a role for chemoradiotherapy in patients with lymph node positive disease with a high lymph node ratio.

### Resection Margin

Most studies that explore the efficacy of postoperative treatment exclusively include patients who underwent a radical resection with D2 lymph node dissection. However, there is also a group of patients in whom the resection is unintentionally not radical (R1). The evidence for managing this patient group is scarce, but a few studies have addressed this question. One of these is a retrospective analysis including data from two phase I/II studies using postoperative chemoradiotherapy, and from the D1D2 trial (104). A total of 785 patients were analyzed of whom 694 patients underwent surgery only. Of the 91 patients who were treated with postoperative chemoradiotherapy, 22 patients underwent an R1 resection; of the 694 patients in the surgery only group, 61 patients underwent an R1 resection. A statistically significant improved 2-year OS was observed in the chemoradiotherapy group (66% vs. 29%. HR 2.91, p=0.002). In another retrospective study, only patients with resectable gastric cancer who were treated with postoperative chemoradiotherapy were studied (105). Out of the 110 patients, 80 patients underwent an R0 resection and 30 patients underwent an R1 resection. Recurrence-free survival and OS were not significantly different between the two groups (p=0.34 and p=0.58 respectively). Although these groups were too small to draw firm conclusions it suggests that patients who underwent R1 resection benefit from postoperative chemoradiotherapy. Postoperative chemoradiotherapy is recommended in the European guidelines for patients who undergo an R1 resection (5). Preferably, the presumed benefit of postoperative chemoradiotherapy should be addressed in a prospective randomized trial. In the meanwhile, subgroup analysis from large randomized trials could be performed, for example from the CRITICS trial.

## Liquid Biopsy

A relatively new topic in gastric cancer is the use of circulating tumor DNA (ctDNA) as a prognostic factor. In order to identify the fraction of ctDNA in cell-free DNA, cell-free DNA is compared to non-malignant DNA from the same patient. In a translational side-study of the CRITICS-trial, the role of ctDNA was investigated among 50 patients by sequencing and analyzing matched cell-free DNA and white blood cell DNA samples (106). The presence of ctDNA at baseline (before start preoperative therapy) was not prognostic for EFS. However, in combination with ctDNA preoperatively, ctDNA was effective for predicting pathological response. Seven responders were identified based on baseline plus preoperative ctDNA, and all of them achieved complete or major pathologic response. On the other hand, three patients were ctDNA negative at baseline, but weeks later had preoperatively detectable ctDNA. These three patients all developed recurrent disease. Preoperative presence of ctDNA was confirmed to be a prognostic factor. Especially the presence of ctDNA at the postoperative time point seemed to be prognostic for survival: after a median follow-up of 42 months, all 11 patients without detectable ctDNA postoperatively were alive and free of recurrence at time of last follow-up. On the other hand, six out of nine patients with detectable ctDNA at the postoperative time point developed disease recurrence.

Another study from China showed comparable results. For 38 patients targeted sequencing analysis of tissue and plasma DNA was performed (107). ctDNA samples were obtained postoperatively, 31 patients were negative for ctDNA, and seven patients were positive for ctDNA. The presence of ctDNA increased the risk for recurrence dramatically: 100% of the patients recurred in the positive group versus 32% in the negative group (p<0.001).

Based on the results of these two studies, it could be concluded that especially the postoperative presence of ctDNA is a very promising new tool to individualize postoperative treatment approaches. Interesting new areas of research would be to investigate whether intensifying treatment in patients with ctDNA present postoperatively would improve survival for this group of patients. It has been postulated that the combination of ctDNA with serological tumor markers could further increase the prognostic value. Classic serological tumor markers in patients with gastric cancer are carcinoembryonic antigen (CEA) and carbohydrate antigen (CA) 19-9. High values of CEA and CA 19-9 have been shown to be negative prognostic factors, as confirmed in large meta-analyses of Asian studies (108, 109). So far, the association between classic serological tumor markers and ctDNA has never been explored.

## Conclusion and Future Perspectives

Currently, most patients with resectable gastric cancer are treated without taking biological variation at the patient and tumor level into account. We believe that future studies should focus on how to individualize treatment. Here, we have provided some directions to consider in these explorations.

Several treatments are currently available, including perioperative chemotherapy, postoperative chemotherapy and pre- and postoperative chemoradiotherapy. It is well-known that more than 50% of patients do not complete postoperative treatment, due to e.g. disease progression, poor condition, preoperative toxicities and postoperative complications (27, 29, 49, 50). In our opinion, future studies should include focus on preoperative treatment. A shift towards (more intensified) preoperative treatment has several advantages, e.g. there are no anatomical distortions, nutritional condition is better, and patients are not recovering from intensive surgery.

In the future, personalization of treatment will be implemented based on patient- and tumor characteristics. Gastric cancer patients form a very heterogeneous group and should not be treated the same. Future trials should use stratifications factors to balance histological and genetically factors. Personalization of treatment will probably lead to intensifying and de-intensifying treatment based on risk of recurrence. Future research is necessary to select treatment (chemotherapy, chemoradiotherapy) for subgroups of gastric cancer patients. Also new therapies are being explored, especially targeted therapy for HER-2 positive tumors and immunotherapy for EBV or MSI-high tumors make a very good chance to be implemented in clinical practice.

In conclusion, many factors affect the tolerability and outcomes. Ideally, future studies would results in a decision tool to identify the optimal treatment for the individual patients taking patient- and tumor characteristics into consideration, as well as prognostic factors known after surgery. Many current and future studies will stimulate steps forward to personalization of treatment in patients with resectable gastric cancer.

## Author Contributions

All authors were involved conception and design of this review. AS drafted the manuscript. MV, EJ, JS, AC, NG, and MV revised this manuscript. All authors contributed to the article and approved the submitted version.

## Conflict of Interest

The authors declare that the research was conducted in the absence of any commercial or financial relationships that could be construed as a potential conflict of interest.
