# Supplementary material for: Towards Personalization in the Curative Treatment of Gastric Cancer
Source: Front Oncol. 2020 Nov 30;10:614907. doi: 10.3389/fonc.2020.614907 (PMC7734340; doi:10.3389/fonc.2020.614907)
Supplement: Supplementary file 1 [file Table_1.docx]

| **Study type** | **Study no. clinicaltrial.gov** | **Title** | **Patient group** | **Design** | **Outcome measures** | **Continent** |
| --- | --- | --- | --- | --- | --- | --- |
| CT | NCT03399110 | XELOX for 4 Months Versus 6 Months in Gastric Cancer (LOMAC) | Stage II-III gastric cancer after resection with D2 LN dissection | ***Arm 1:*** postoperative oxaliplatin plus capecitabine during 6 months  ***Arm 2:*** postoperative oxaliplatin plus capecitabine during 4 months | RFS, adverse events, OS | Asia |
| CT | NCT03941561 | S-1 for 9 Months Versus 1 Year for Stage II Gastric Cancer (SMAC) | Stage II gastric cancer after resection with D2 LN dissection | ***Arm 1:*** postoperative S-1 for 1 year  ***Arm 2:*** postoperative S-1 for 9 months | RFS, OS, adverse events | Asia |
| CT | NCT02555358 | Three Drugs in Advanced Gastric Cancer Neoadjuvant Chemotherapy for Stage Ⅲ Clinical Study | Operable advanced gastric cancer | ***Arm 1:*** preoperative 4 cycles of docetaxel, oxaliplatin and capecitabine, postoperatively 4 cycles of capecitabine and oxaliplatin  ***Arm 2:*** preoperative 4 cycles of capecitabine and oxaliplatin, postoperatively 4 cycles of capecitabine and oxaliplatin  ***Arm 3:*** postoperative 8 cycles of capecitabine and oxaliplatin | pCR rate, OS, PFS, RFS, adverse events | Asia |
| CT | NCT01917552 | Adjuvant Capecitabine Versus Observation Alone in Curatively Resected Stage IB Gastric Cancer((KCSG ST14-05): CATALYSIS | Stage IB gastric cancer | ***Arm 1:*** observation  ***Arm 2:*** postoperative capecitabine (duration not specified) | RFS, OS, adverse events | Asia |
| CT | NCT02867839 | Adjuvant Chemotherapy With S-1 Plus Oxaliplatin Versus S-1 Alone in Locally Advanced Gastric Cancer (RESCUE-GC) | Stage II/IIIA gastric cancer after resection with D2 LN dissection | ***Arm 1:*** postoperative 8 cycles of oxaliplatin plus S-1 (6 months)  ***Arm 2:*** postoperative 16 cycles of S-1 (12 months) | RFS, OS, adverse events | Asia |
| CT | NCT03788226 | A Randomized Phase III Study Comparing POF With SOX/CAPOX/FOLFOX as a Postoperative Adjuvant Chemotherapy for Curatively Resected Stage III Gastric Cancer | Stage III gastric cancer after resection and D2 LN dissection | ***Arm 1:*** standard postoperative CT. Capecitabine and oxaliplatin for 8 cycles, OR S-1 and oxaliplatin for 8 cycles, OR 5-FU, leucovorin and oxaliplatin for 12 cycles.  ***Arm 2:*** postoperative paclitaxel, oxaliplatin and leucovorin for 12 cycles | 3-year RFS, OS, adverse events | Asia |
| CT | NCT03322969 | Receiving Modified Chemotherapy Followed With Radical Resection After Neoadjuvant Chemotherapy | Stage T2-T4 and/or N+, M0 gastric cancer | All patients received preoperative chemotherapy followed by resection.  ***Arm 1:*** postoperative capecitabine and oxaliplatin OR S-1 and oxaliplatin.  ***Arm 2:*** postoperative paclitaxel /DDP  Schedules not specified | RFS | Asia |
| CT | NCT02512380 | Neoadjuvant SLOT Versus SOX in Patients With Locally Advanced, Resectable Gastric/Esophagogastric Junction (EGJ) Cancer | Stage T3-T4, N0/N+, MO gastric cancer | ***Arm 1:*** perioperative 4 cycles of S-1 and oxaliplatin.  ***Arm 2:*** preoperative 4 cycles of docetaxel, oxaliplatin and S-1. Postoperative 6 cycles of S-1 plus oxaliplatin, followed by S-1 monotherapy for 6 months | OS, RFS, R0 rate, response, quality of life | Asia |
| CT | NCT03817268 | Capecitabine or Observation for Patients With pT1N+M0 or pT2-3N0M0 Gastric Adenocarcinoma Undergoing R0 Resection (CAPOGA) | Stage T1, N+, M0 or T2-3, N0, M0 who underwent resection plus D2 LN dissection | ***Arm 1:*** no intervention  ***Arm 2:*** postoperative capecitabine monotherapy for 8 cycles | RFS, OS, adverse events, quality of life | Asia |
| CT | NCT03607656 | The Effect of Traditional Chinese Treatment Combined Adjuvant Chemotherapy in IIIb and IIIc Gastric Cancer (CHANGE) | Stage IIIB or IIIC gastric cancer, after resection with D2 LN dissection | ***Arm 1:*** postoperative capecitabine and oxaliplatin OR S-1 and oxaliplatin, OR docetaxel and S-1, OR docetaxel and oxaliplatin and 5-FU. All combinations of chemotherapy for 8 cycles.  ***Arm 2:*** traditionally Chinese medicine (TCM) combined with the chemotherapy in arm 1 | 3-year RFS, adverse events, quality of life | Asia |
| CT | NCT04135781 | Nab-paclitaxel Combined With S-1 as Adjuvant Chemotherapy for Stage III Gastric Cancer | Stage III gastric cancer after resection plus D2 LN dissection | ***Arm 1:*** postoperative capecitabine and oxaliplatin for 8 cycles  ***Arm 2:*** nab-paclitaxel and S-1 for 8 cycles | 3-year RFS, OS, adverse-events | Asia |
| CT | NCT01935778 | Compare Adjuvant Chemotherapy of Docetaxel/Capecitabine/Oxliplatin Versus Capecitabine/Oxaliplatin in Advanced Gastric Cancer at Stage IIIb and IV(KCSG ST15-08): TRIUMPH | Stage III/IV (M0) gastric cancer after resection plus D2 LN dissection | ***Arm 1:*** postoperative capecitabine and oxaliplatin (duration not specified)  ***Arm 2:*** postoperative docetaxel, oxaliplatin and capecitabine (duration not specified) | RFS, OS, adverse events | Asia |
| CT | NCT01665274 | Efficiency of XELOX Neoadjuvant Chemotherapy in Gastric Cancer | Stage cT2-4N+M0 and cT4N0M0 gastric cancer | ***Arm 1:*** postoperative capecitabine and oxaliplatin for 6 cycles  ***Arm 2:*** preoperative 3 cycles of capecitabine and oxaliplatin. Postoperative capecitabine and oxaliplatin for 3 cycles (if CR or PR) | RFS, adverse events, response rate, R0 rate, OS | Asia |
| CT/CRT | NCT01815853 | Neoadjuvant Chemoradiotherapy vs. Chemotherapy With Radical Gastrectomy and Adjuvant Chemotherapy for Advanced Gastric Cancer (Neo-CRAG) | cT3N2/N3M0, cT4aN+M0 or cT4bNanyM0 gastric cancer | ***Arm 1:*** perioperative 3 cycles of capecitabine and oxaliplatin  ***Arm 2:*** preoperative radiotherapy (45Gy) plus 3 cycles of capecitabine and oxaliplatin. Postoperative 3 cycles of capecitabine and oxaliplatin | RFS, OS, response, R0 rate, adverse events | Asia |
| CT/CRT | NCT03223740 | Preoperative Stomach Cancer Induction Chemotherapy and Radiation Therapy (President) | cT3-4, any N, M0 gastric cancer | ***Arm 1:*** postoperative capecitabine and oxaliplatin for 2 cycles, followed by chemoradiotherapy (45 Gy plus capecitabine and taxol), followed by 2 cycles of capecitabine  ***Arm 2:*** preoperative capecitabine and oxaliplatin for 2 cycles, followed by chemoradiotherapy (45 Gy plus capecitabine and taxol), followed by 2 cycles of capecitabine | PFS, OS, R0 rate | Asia |
| CT/CRT | NCT03601988 | Phase III Trial of Adjuvant Chemo-radiotherapy After D2 Surgery of Gastric Cancer | Stage T4/N2/N3 gastric cancer after resection with D2 LN dissection | ***Arm 1:*** postoperative 8 cycles of oxaliplatin and capecitabine  ***Arm 2:*** postoperative 6 cycles of oxaliplatin and capecitabine plus concurrent chemoradiotherapy (45 Gy plus capecitabine) | RFS, OS | Asia |
| CT/CRT | NCT03013010 | PREACT Study: Locally Advanced Gastric Cancer, Chemoradiotherapy vs. Chemotherapy Followed by D2 Surgery and Adjuvant Chemotherapy (PREACT) | Stage IIB-III gastric cancer | ***Arm 1:*** perioperative 3 cycles of S-1 and oxaliplatin  ***Arm 2:*** preoperative 1 cycle of S-1 and oxaliplatin, followed by 5 weeks of chemoradiotherapy (45 Gy in 25 fractions plus S-1), followed by 1 cycle of S-1 plus oxaliplatin. Postoperative 3 cycles of S-1 and oxaliplatin | RFS, OS, pCR rate, adverse events, postoperative complications | Asia |
| CT/CRT | NCT01924819 | Trial of Preoperative Therapy for Gastric and Esophagogastric Junction Adenocarcinoma (TOPGEAR) | Stage IB (T1N1 only, T2N0 not eligible) - IIIC, i.e. T3 - T4 and/or node positive gastric cancer | ***Arm 1:*** perioperative 3 cycles of epirubicin, cisplatin and 5-fluorouracil OR epirubicin, cisplatin and capecitabine OR epirubicin, oxaliplatin and capecitabine OR 5-Fluorouracil, leucovorin, oxaliplatin and docetaxel (last schedule 4 cycles)  ***Arm 2:*** preoperative 2 cycles of chemotherapy (schedules mentioned at arm 1), followed by chemoradiotherapy (45 Gy plus 5-FU or capecitabine). Postoperative similar to arm 1. | OS, RFS, response, adverse events, R0 rate | Australia, Europe, Northern America |
| CT | NCT01787539 | The Role of Postoperative Cycles in the Perioperative Chemotherapy for Gastric Cancer (STOPEROPCHEM) | Stage cT24a-N0-3M0 gastric cancer | ***Arm 1:*** preoperative epirubicin, oxaliplatin and capecitabine, no postoperative therapy.  ***Arm 2:*** preoperative epirubicin, oxaliplatin and capecitabine. In case of regression grade 0, 1 or 2, patients will receive postoperative epirubicin, oxaliplatin and capecitabine. | RFS, OS, adverse events, grade 5 toxicity, dose reductions, quality of life | Europe |
| CT/CRT | NCT02931890 | Multicentric Randomised Trial for Resectable Gastric Cancer (CRITICS-II) | Stage IB-IIIC gastric cancer | ***Arm 1:*** preoperative 4 cycles of docetaxel, oxaliplatin and capecitabine  ***Arm 2:*** preoperative 2 cycles of docetaxel, oxaliplatin and capecitabine, followed by chemoradiotherapy (45 Gy in 25 fraction, combined with paclitaxel and carboplatin)  ***Arm 3:*** preoperative chemoradiotherapy (45 Gy in 25 fraction, combined with paclitaxel and carboplatin) | EFS, adverse events | Europe |
| CT | NCT04393584 | FOLFIRINOX vs FLOT Chemotherapy for Resectable Gastric or Esophagogastric Junction Adenocarcinoma (RusGCG-01) | cT4N0 or cT1-4N+, M0 GEJ or gastric cancer | ***Arm 1:*** perioperative 4 cycles of irinotecan, leucovorin, 5-FU and oxaliplatin for 4 cycles.  ***Arm 2***: pre- and postoperative 4 cycles of FLOT | OS, response, RFS, perioperative morbidity and mortality, R0 rate | Russia |

***Supplementary Table 1:*** Running randomized phase II-III studies in patients with resectable gastric cancer, subdivided by type of therapy. Abbreviations: CT = chemotherapy, CRT = chemoradiotherapy, LN= lymph node, OS= overall-survival, RFS= recurrence-free survival/disease-free survival, R0= resection margin 0 (clear margins), pCR= pathologic complete response, FLOT= fluorouracil, leucovorin, oxaliplatin, docetaxel
