# Supplementary material for: Towards Personalization in the Curative Treatment of Gastric Cancer
Source: Front Oncol. 2020 Nov 30;10:614907. doi: 10.3389/fonc.2020.614907 (PMC7734340; doi:10.3389/fonc.2020.614907)
Supplement: Supplementary file 2 [file Table_2.docx]

| **New therapy** | **Study no. clinicaltrial.gov** | **Title** | **Patient group** | **Design** | **Outcome measures** | **Continent** |
| --- | --- | --- | --- | --- | --- | --- |
| IT | NCT04354662 | Toripalimab Combined With FLOT Neoadjuvant Chemotherapy in Patients With Resectable Gastric Cancer | Stage (>T1) and/or nodal positive (N+) gastric cancer | Perioperative 4 cycles of FLOT plus toripalimab | 3-year DFS, response, adverse-events, 5-year RFS, 5-year OS | Asia |
| IT | NCT04515615 | Adjuvant Chemotherapy in Combination With Camrelizumab for Stage III Gastric Cancer (FOCUS-02) | Stage III gastric cancer after resection with D2 LN dissection | Postoperative 8 courses of camrelizumab, oxaliplatin and tagafur-gimeracil-oteraxil-potassium capsule | Adverse events, RFS, OS, treatment compliance | Asia |
| TKI, IT | NCT03878472 | Neoadjuvant Immunotherapy for Resectable Gastric Cancer | Stage T3-4aN+M0 with proximal gastric cancer | ***Arm 1:*** preoperative SHR1210  ***Arm 2:*** preoperative SHR1210 + apatinib  ***Arm 3:***  preoperative SHR1210 + apatinib + S-1  ***Arm 4:***  preoperative SHR1210 + apatinib + S-1 + oxaliplatin | pCR rate, ORR, PFS, OS, adverse events, R0 rate, response | Asia |
| IT | NCT03006705 | Study of Adjuvant ONO-4538 With Resected Gastric Cancer | Stage III gastric cancer after resection with D2 LN dissection | ***Arm 1:*** postoperative S-1 (maximum 1 year) or capecitabine plus oxaliplatin (maximum 6 months), up to the investigator  ***Arm 2:*** postoperative S-1 (maximum 1 year) or capecitabine plus oxaliplatin (maximum 6 months). Both options plus nivolumab | RFS, OS, adverse events | Asia |
| IT | NCT04139135 | A Clinical Study of HLX10 Combined With Chemotherapy Versus Placebo Combined With Chemotherapy for Neoadjuvant/Adjuvant Treatment of Gastric Cancer | PD-L1 positive gastric cancer, stage not specified | ***Arm 1:*** preoperative S-1 and oxaliplatin for three cycles. Postoperative S-1 and oxaliplatin for 5 cycles  ***Arm 2:*** preoperative S-1, oxaliplatin and HLX10 for 3 cycles. Postoperative HLX10 for 17 cycles | EFS, RFS, pCR, OS | Asia |
| IT, TKI | NCT04195828 | Camrelizumab Combined With Apatinib Mesylate Tablets, Nab-paclitaxel and S-1 in the Treatment of Locally Advanced Gastric Cancer | Stage cT2-4a and/or N+, M0 gastric cancer | ***Arm 1:*** preoperative cemrelizumab combined with apatinib, nab-paclitaxel and S-1  ***Arm 2:*** preoperative nab-paclitaxel and S-1 | pCR rate, ORR, R0 resection rate | Asia |
| IT | NCT04152889 | A Study to Evaluate Camrelizumab in Combination With Docetaxel +S-1 as Adjuvant Treatment Therapy in Stage III Gastric Cancer | Stage III Gastric Cancer (PD-L1 + / MSI / EBV +/dMMR) | Camrelizumab plus S-1 and docetaxel for 7 cycles | RFS | Asia |
| IT | NCT04250948 | Efficacy of Perioperative Chemotherapy Plus PD-1 Antibody in the Locally Advanced Gastric Cancer | Stage cT3-4aN+M0 gastric cancer | ***Arm 1:*** capecitabine and oxaliplatin OR S-1 and oxaliplatin. Preoperative for 3 cycles, postoperative for 5 cycles  ***Arm 2:*** same type of chemotherapy as arm 1, combined with JS001 | Response, R0 rate, RFS, OS | Asia |
| TKI | NCT03229096 | Apatinib Plus XELOX as Neoadjuvant Therapy in Locally Advanced Gastric Cancer | Stage c T2-T4aN+M0 gastric cancer | Preoperative 4 cycles of capecitabine and oxaliplatin plus apatinib during the first 3 cycles. | ORR, adverse events, response, RFS, OS | Asia |
| IT | NCT04119622 | Toripalimab Combined With Chemotherapy as Neoadjuvant Treatment of Gastric Cancer | Stage II/III gastric cancer | Preoperative 2 cycles of toripalimab plus capecitabine and oxaliplatin. After 2 cycles response assessment. If CR/PR/SD surgery will be performed. In case of PD patients could receive one additional cycle, or chemoradiotherapy, or another type of chemotherapy | RR, adverse events, RFS, OS | Asia |
| IT | NCT04202601 | Efficacy and Safety Evaluation of Sintilimab in Combination With IBI310 as Treatment in Patients With EBV-Positive Gastric Cancer | One neoadjuvant group: Stage T3-4aN0M0 or T2-T4, N+, M0 gastric cancer | Perioperative sintilimab + IBI310 for 3 cycles, postoperatively followed by 4 cycles of sintilimab monotherapy | Response, EFS, OS, adverse events | Asia |
| IT | NCT04065282 | The Purpose of This Study is to Evaluate the Efficacy and Safety of Sintilimab in Combination With Xelox as Neoadjuvant Therapy for Patients With Resectable Locally Advanced Gastric or Gastroesophageal Adenocarcinoma. | Stage T3-4, any N, M0 gastric cancer | Preoperative sintilimab plus capecitabine and oxaliplatin for 3 cycles | Response, RFS, OS | Asia |
| TT | NCT03950271 | SHR-1210 Combined With Trastuzumab , Oxaliplatin and Capecitabine for Neoadjuvant Therapy of Gastric Adenocarcinoma/Gastroesophageal Junction Adenocarcinoma | HER2 positive gastric cancer stage T4 and/or N+, M0 | Preoperative 4 cycles of SHR-1210, trastuzumab, and capecitabine. Postoperative 8 cycles of capecitabine and oxaliplatin | RFS, ORR, OS, pCR rate | Asia |
| IT | NCT03979131 | Phase II Study of Avelumab Plus Chemotherapy in the Peri-operative Treatment for Patients With Resectable Gastric Cancer (GC) or Gastroesophageal Junction Cancer (GEJC) (MONEO) | Stage IB-IIIC gastric cancer | Perioperative FLOT plus avelumab for 4 cycles. Postoperative 4 cycles of the same regimen, followed by avelumab treatment up to 1 year. | Response, OS, RFS, PFS, R0 rate | Europe |
| IT | NCT03421288 | Phase II Study of Atezolizumab + FLOT vs. FLOT Alone in Patients With Gastric Cancer and GEJ (DANTE) | Stage (cT2, cT3, cT4, any N category,  M0), or (any T, N+, M0) gastric cancer | ***Arm 1:*** perioperative FLOT for 4 cycles  ***Arm 2:*** preoperative FLOT plus atezolizumab for 4 cycles, postoperative FLOT plus atezolizumab for 4 cycles followed by 8 cycles of atezolizumab alone | RFS/PFDS, response, R0 rate, OS, immune cell inflammation rate | Europe |
| IT | NCT04062656 | Perioperative Chemotherapy vs Immunotherapy vs. Chemo-immunotherapy in Patients With Advanced GC and AEG (IMAGINE) | cT2-T4, any N, M0 gastric cancer | ***Arm 1:*** perioperative FLOT 4 cycles  ***Arm 2:*** perioperative FLOT in combination with nivolumab  ***Arm 3:*** perioperative FLOT in combination with nivolumab and ipilimumab  ***Arm 4:*** perioperative FLOT in combination with nivolumab and relatlimab | Response, R0 rate, OS, adverse events, perioperative morbidity and mortality, RFS, quality of life | Europe |
| TT | NCT02205047 | Neoadjuvant Study Using Trastuzumab or Trastuzumab With Pertuzumab in Gastric or Gastroesophageal Junction Adenocarcinoma (INNOVATION) | HER2 positive gastric cancer stage | ***Arm 1:*** preoperative 3 cycles of cisplatin/capecitabine or cisplatin/5-FU  ***Arm 2:*** preoperative 3 cycles of cisplatin/capecitabine plus trastuzumab or cisplatin/5-FU plus trastuzumab  ***Arm 3:*** preoperative 3 cycles of cisplatin/capecitabine plus trastuzumab and pertuzumab or cisplatin/5-FU plus trastuzumab and pertuzumab | Near complete response, RFS, R0 rate, PFS, OS, adverse events | Europe |
| IT | NCT03443856 | Postoperative Immunotherapy vs Standard Chemotherapy for Gastric Cancer With High Risk for Recurrence (VESTIGE) | Stage Ib-IVa gastric and esophagogastric junction adenocarcinoma and high risk of recurrence (defined by ypN1-3 and/or R1 status) | ***Arm 1:*** perioperative chemotherapy according ESMO guidelines  ***Arm 2:*** postoperative nivolumab plus ipilimumab for 4 cycles (3 months) followed by nivolumab monotherapy for 9 months | RFS, OS, local failure rates, adverse events, quality of life | Europe |
| IT | NCT03448835 | Neoadjuvant Capecitabine, Oxaliplatin, Docetaxel and Atezolizumab in Resectable Gastric and GE-junction Cancer (PANDA) | Resectable GEJ or gastric carcinoma | Preoperative 1 cycle of atezolizumab followed by 4 cycles atezolizumab, capecitabine, oxaliplatin and docetaxel | Adverse events, response | Europe |
| IT | NCT03399071 | Peri-operative Immuno-Chemotherapy in Operable Oesophageal and Gastric Cancer (ICONIC) | Stage T1-3, N0-2, M0 gastric cancer | Perioperative FLOT plus Avelumab for 4 cycles | Response, adverse events, PFS, OS | UK |
| IT | NCT02918162 | Perioperative Chemo and Pembrolizumab in Gastric Cancer | cT2 and/or N+, M0 gastric cancer | Perioperative chemotherapy with at least a platinum and fluorouracil agent, 3 cycles, combined with pembrolizumab. | RFS, response, OS, ORR | US |
| IT | NCT03257163 | Pembrolizumab, Capecitabine, and Radiation Therapy in Treating Patients With Mismatch-Repair Deficient and Epstein-Barr Virus Positive Gastric Cancer | MMRD and EBV positive gastric cancer stage T2-T4, N0-N3, M0 | Pembrolizumab for two courses, followed by surgery, followed by postoperative pembrolizumab and capecitabine for 5 courses, within 2-6 weeks of rest, start of pembrolizumab up to 11 courses. Beginning of course 4, start radiotherapy on day 1-5 for 5 weeks. | RFS | US |
| IT | NCT03776487 | Nivolumab, Ipilimumab and Chemoradiation in Treating Patients With Resectable Gastric Cancer | Until stage IVa gastric cancer (resectable, M0) | Preoperative oxaliplatin and 5-FU for 4 courses, followed by nivolumab up to 6 courses. Beginning of course 4, patients receive 5-FU combined with radiotherapy 45 Gy in 25 fractions. Patients with residual disease after surgery are treated with nivolumab for 8 courses, then every 4 weeks up to 2 courses in absence of disease progression or unacceptable toxicity | Adverse events, response rate, RFS | US |
| IT | NCT03064490 | Pembrolizumab, Radiotherapy, and Chemotherapy in Neoadjuvant Treatment of Malignant Esophago-gastric Diseases (PROCEED) | Patients with resectable GEJ of gastric carcinoma, intention to treat with preoperative chemoradiotherapy | Preoperative 3 doses of pembrolizumab. Pembrolizumab is combined with weekly standard of care (carboplatin/paclitaxel) concurrent with radiotherapy. Postoperative 3 courses of pembrolizumab | pCR, adverse events | US |
| IT | NCT03221426 | Study of Pembrolizumab (MK-3475) Plus Chemotherapy Versus Placebo Plus Chemotherapy in Participants With Gastric or Gastroesophageal Junction (GEJ) Adenocarcinoma (MK-3475-585/KEYNOTE-585) | T3 or greater and/or node positive gastric cancer, M0 | ***Arm 1:*** preoperative 3 cycles of pembrolizumab plus cisplatin and capecitabine or 5-FU. Postoperative pembrolizumab plus cisplatin and capecitabine or 5-FU.  ***Arm 2***: preoperative 3 cycles of placebo plus cisplatin and capecitabine or 5-FU. Postoperative 3 cycles of preoperative placebo plus cisplatin and capecitabine or 5-FU.  ***Arm 3:*** preoperative 3 cycles of pembrolizumab plus 4 cycles of FLOT. Postoperative 11 cycles of pembrolizumab plus 4 cycles of FLOT.  ***Arm 4:*** preoperative 3 cycles of placebo plus 4 cycles of FLOT. Postoperative 11 cycles of pembrolizumab plus 4 cycles of FLOT. | OS, EFS, pCR rate, adverse events, RFS, OS | US |
| IT | NCT03488667 | Perioperative mFOLFOX Plus Pembrolizumab in Gastroesophageal Junction (GEJ) and Stomach Adenocarcinoma | Potentially resectable gastric cancer, M0 | Preoperative 3 cycles of pembrolizumab plus 4 cycles of fluorouracil and oxaliplatin. Postoperative 3 cycles of pembrolizumab plus 4 cycles of fluorouracil and oxaliplatin. | Response rate, adverse events, RFS, OS, PET response, PD-L1 expression | US |
| IT | NCT02730546 | Pembrolizumab, Combination Chemotherapy, and Radiation Therapy Before Surgery in Treating Adult Patients With Locally Advanced Gastroesophageal Junction or Gastric Cardia Cancer That Can Be Removed by Surgery | Patients with T2-T4, and/or N+, M0 gastric cancer | Preoperative 4 cycles of pembrolizumab, leucovorin, fluorouracil OR pembrolizumab plus paclitaxel and carboplatin combined with radiotherapy for 4 weeks and 4 days in 23 fractions. | pCR rate, PDS, R0 rate, RFS, adverse events, surgical complications, OS | US |
| IT, TT | NCT04510285 | Study of Pembrolizumab Plus Trastuzumab or Trastuzumab Alone After Surgery in Patients With Esophagogastric Tumors | Patients with resected esophageal or GEJ or gastric cancer who underwent resection, must have completed standard of care (preoperative or postoperative therapy) | ***Arm 1***: postoperative trastuzumab (number of cycles not defined)  ***Arm 2:*** postoperative trastuzumab plus pembrolizumab (number of cycles not defined) | ctDNA clearance at 6 months follow-up. | US |

***Supplementary Table 2:*** Running studies exploring the role of ´new therapies´ (immunotherapy, targeted therapy tyrosine kinase inhibitors), all phases. Abbreviations: LN= lymph node, OS= overall-survival, RFS= recurrence-free survival/disease-free survival, R0= resection margin 0 (clear margins), pCR= pathologic complete response, FLOT= fluorouracil, leucovorin, oxaliplatin, docetaxel, IT= immunotherapy, TT=targeted therapy, TKI= tyrosine kinase inhibitor
